# Nutritional and Functional Fortification of Cultivated Meat: A Scoping Review

**DOI:** 10.3390/foods15162840

**Published:** 2026-08-14

**Authors:** Cheuk Lun Wong, Xinyue Liu, Jing Guo, Federica Cheli, Carlotta Giromini

**Affiliations:** 1 Department of Veterinary and Animal Sciences (DIVAS), Università degli Studi di Milano, Via dell’Università 6, 29600 Lodi, Italy; federica.cheli@unimi.it (F.C.); carlotta.giromini@unimi.it (C.G.); 2 School of Food Science and Nutrition, Faculty of Environment, University of Leeds, Leeds LS2 9JT, UK; fs23xl@leeds.ac.uk (X.L.); s.j.guo@leeds.ac.uk (J.G.); 3 Institute for Food, Nutrition and Health, University of Reading, Reading RG6 6EU, UK

**Keywords:** cellular agriculture, cell culture, cultivated meat, fortification, novel food, nutrients

## Abstract

With the ever-growing population and increasing demand for food, cultivated meat (CM) represents a potentially sustainable alternative source of protein. However, one of the critical challenges is ensuring that the nutritional value of CM is comparable to that of conventional meat. In light of this, this scoping review aimed to systematically map the existing evidence from in vitro studies and identify potential nutritional fortification methods for CM. Literature searches were conducted in PubMed, Embase, and Web of Science from inception to December 2025, supplemented by a hand search in January 2026. Studies were included if they were in vitro studies related to cultivated food that applied a fortification strategy or treatment to improve the nutritional value or food functionality and reported the relevant outcomes. Extracted data (e.g., fortification methods, cell lines, study results) were synthesized descriptively. Seven studies were eligible for inclusion. Three fortification strategies were identified, including metabolic engineering, product reformulation, and nutrient-enriched cell culture medium. Reported outcomes varied among the included studies and included changes in nutritional value, food functionality, and cellular functionality of CM. However, most studies used cell models that are primarily intended for research purposes rather than food production or human consumption, which may limit their relevance to commercial CM products. In conclusion, this review identified preliminary fortification approaches that may modify the nutritional value, food functionality, or cellular functionality of CM. However, further investigation is needed to determine their effects on the nutritional composition of the resulting cell biomass or final CM product.

## 1. Introduction

Cultivated meat (CM) has been proposed as a potentially sustainable alternative to meet the increasing demand for food. More importantly, CM may also offer opportunities for nutritional optimization, such as increasing nutritionally beneficial compounds or reducing saturated fat content, rather than simply replicating the nutritional profile of conventional meat products.

**Figure 1:**
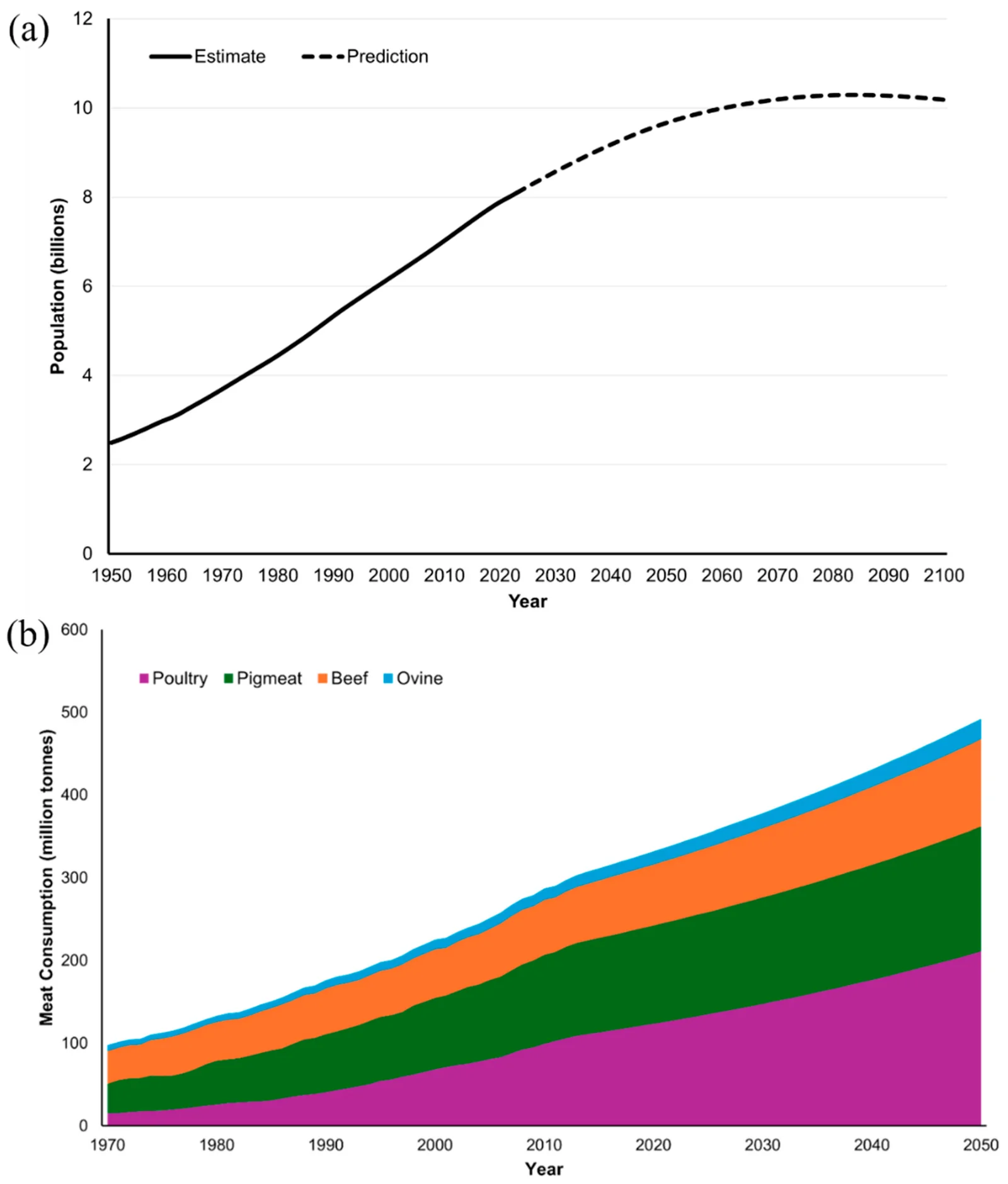
(**a**) Global population growth from 1950 to 2100. The solid line represents observed population estimates, while the dotted line represents projected population growth from 2020 onwards [1]. (**b**) Global meat consumption from 1970 to 2050. Data from 1970 to 2013 were based on the Food and Agriculture Organization of the United Nations (FAO) estimates, whereas data from 2013 to 2050 were based on FAO projections [3].

**Figure 2:**
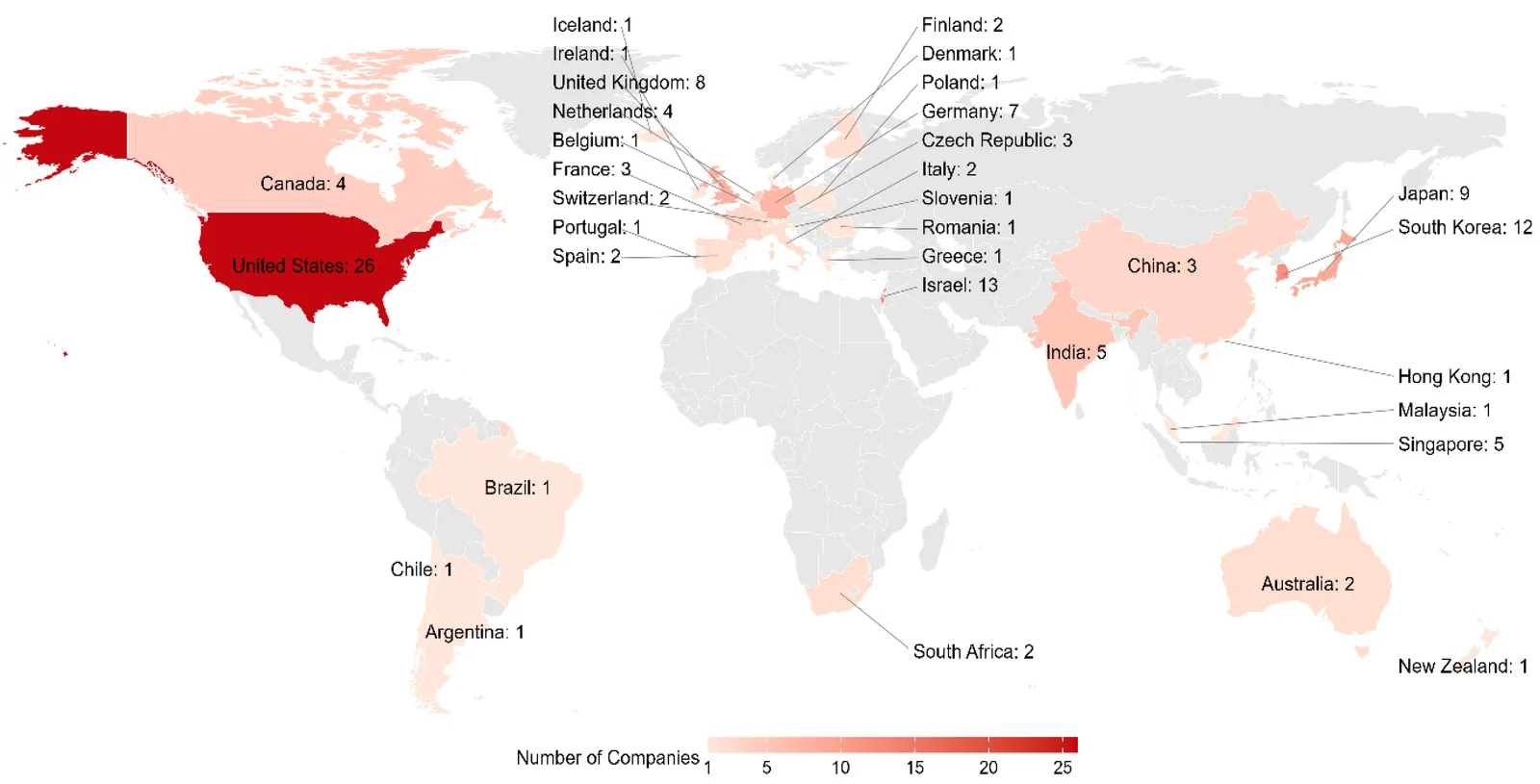
Global distribution of cultivated meat and pet food companies as of 2026. The geographical spread of these companies reflects commercial interest in cellular agriculture. Data from Good Food Institute [8].

The global population is projected to reach 9–11 billion (Figure 1a), while global meat consumption is estimated to reach approximately 500 million tonnes by 2050 (Figure 1b) [1,2,3]. Consequently, interest in CM research has grown considerably in recent years [4], as CM has been discussed as one possible way to address food security challenges. In addition, reflecting growing commercial interest in CM, as of 2026, more than one hundred companies around the world are working on cultivated meat and pet food (Figure 2). Given the continued expansion of the market and ongoing product development, the nutritional composition and quality of CM require greater attention. However, CM remains controversial due to ongoing debates on its actual feasibility and sustainability. In fact, there are still many challenges faced by CM, including biotechnological constraints (e.g., cell proliferation, nutritional and functional properties), technical difficulties (e.g., industrial process scale-up), and regulatory barriers (e.g., market approval) [5,6,7].

Beyond its potential as an alternative protein source, CM has the potential to provide a nutritional value comparable to, or even better than, that of conventional meat [9,10], which could benefit consumers’ health and address their concerns. It is crucial that the nutritional value of CM is comparable to conventional meat, given that meat is rich in easily digestible proteins, amino acids, vitamins and minerals [11], as the core idea of CM is to mimic conventional meat. It has been known for a long time that red meat and processed meat are rich in saturated fatty acids (SFAs), which are positively associated with the risk of chronic diseases, such as cardiovascular disease and colorectal cancer [12]. However, the nutritional composition of CM, for example, the fat composition, could potentially be optimized through controlled cultivation conditions, supplementation of the culture medium, and co-culturing with adipose cells [10]. With these strategies, the production of CM with reduced SFA content compared to red and processed meat becomes possible [5].

Evidence on consumer acceptance has highlighted concerns about the naturalness, healthiness, food safety and nutritional value of CM compared to conventional meat [13]. These factors strongly influence willingness to consume (WTC) CM [14]. Therefore, addressing these concerns is essential for facilitating the broader adoption of CM. However, the nutritional profile of CM remains incompletely characterized, particularly for micronutrients. In conventional meat, nutrients accumulate in muscle tissue through the digestion of animal feed. For example, vitamin B12 can be produced in ruminants by rumen microorganisms and then the synthesized vitamin B12 is absorbed and stored in liver and muscle [15], contributing to the nutritional content of conventional meat. In contrast, CM lacks this biological mechanism. CM may require strategies to achieve a desirable nutritional profile [11]. Therefore, investigating strategies to fortify CM is essential.

To date, the concept of nutritional fortification has not yet been standardized in the context of cultivated meat. In general, food fortification (i.e., direct fortification) refers to the addition of essential vitamins and minerals to foods during processing in order to improve nutritional quality and provide public health benefits [16]. The production of CM involves multiple stages, for example, muscle stem cell isolation, proliferation and differentiation of muscle stem cells, scaffold seeding, and tissue maturation [6]. This indicates that the nutritional profile of CM could be modified or enriched at different stages of production. Therefore, clear definitions are needed to distinguish among the different approaches used to modify the nutritional value, food functionality, or cellular functionality of CM. In this review, the term ‘nutritional fortification strategies’ is used as an umbrella term for several possible approaches, including genetic or metabolic engineering, product reformulation, cell culture medium enrichment, and conventional post-harvest fortification. These different approaches may have different scientific and regulatory implications because they differ in their biological mechanisms and stages of application.

Despite the growing development and use of cell-based food technology in several countries, relatively few studies have investigated the nutritional fortification strategies for CM, and reviews synthesizing this evidence remain scarce. Given the importance of ensuring the nutritional value of CM and the lack of a comprehensive review on CM fortification, this scoping review aimed to systematically synthesize the existing evidence of in vitro studies related to CM fortification. This review outlined current fortification strategies and highlighted key research gaps. To our knowledge, it is the first to systematically map in vitro fortification strategies for CM and to inform future research in this area.

## 2. Materials and Methods

This scoping review was reported in accordance with the Preferred Reporting Items for Systematic Reviews and Meta-Analyses extension for Scoping Reviews (PRISMA-ScR) [17], and follows the guidance of the Joanna Briggs Institute (JBI) scoping review [18]. The protocol of this scoping review was registered in the Open Science Framework (OSF) (https://doi.org/10.17605/OSF.IO/X6243) on 13 February 2026. The PRISMA-ScR checklist is presented in the Supplementary Materials.

### 2.1. Eligibility Criteria

The inclusion criteria were developed based on the framework of Population, Concept, and Context (PCC) recommended by the JBI scoping review guidance [18], in which ‘Population’ referred to the cell types used in cultivated food (CF) research, including established cell lines and primary cells; ‘Concept’ referred to the fortification strategies intended to enhance nutritional value, food functionality, or cellular functionality of CF, and ‘Context’ was restricted to in vitro studies related to CF research. The term ‘nutritional value’ referred to measurable changes in nutrient content or composition, such as proteins, vitamins, minerals, and other nutritionally relevant compounds. ‘Food functionality‘ referred to parameters related to antioxidant capacity and sensory properties, whereas ‘cellular functionality’ referred to cell proliferation, differentiation, and viability. Only peer-reviewed journal in vitro studies were eligible for inclusion, and no language restriction was applied. The inclusion and exclusion criteria were as follows: (1) in vitro studies regardless of the study purpose and the cell line used, (2) studies that were related to CF, (3) studies that reported applied treatment or fortification method that might improve the nutritional value, food functionality, or cellular functionality of the CF, and (4) studies that reported the change in nutritional content in relation to the fortification, food functionality, or cellular functionality of the CF. Studies were excluded if they were (1) duplicate records, (2) not in vitro studies, (3) not related to CF, (4) did not apply any treatment that aimed at improving nutritional or functional properties of the CF, or (5) did not report measurable nutritional or functional outcomes.

### 2.2. Search Strategy

The search strategy was developed based on the PCC framework mentioned above. The literature search was conducted in December 2025 through three databases, including PubMed, Embase and Web of Science. The literature from inception to the date of the search conducted was included without language restriction. To ensure comprehensive coverage, supplementary hand searching of relevant reference lists and key authors, including authors/coauthors of our research team, was performed in January 2026. The keywords for the literature search were combined with Medical Subject Headings (MeSH), and the details of the keywords are presented in Table S1.

### 2.3. Study Selection

The study selection was conducted independently by two researchers (C.L.W. and X.L.). The procedures included removing duplicates, followed by screening titles and abstracts of studies based on whether the studies were relevant to in vitro experiments and CF. Then, the full texts of studies that met the inclusion criteria were retrieved and screened based on the predefined eligibility criteria described above. Any disagreements regarding study eligibility were resolved by the researchers through discussion and consensus. To minimize bias caused by potential overlapping authorship (i.e., the inclusion of studies related to the authors/coauthors of our research team), the screening process was strictly based on predefined criteria and conducted independently by the two researchers.

### 2.4. Data Extraction

Key information of the included studies was extracted by one researcher (C.L.W.) and then independently verified by a second researcher (X.L.). Any discrepancies were discussed and resolved among the researchers through discussion and consensus. Information from the eligible studies was extracted, including (1) author and publication year, (2) cell type, (3) passage number of the cells, (4) description of the fortification method, (5) conditions of the control groups, (6) proliferation and differentiation duration, (7) findings of the studies, and (8) components of proliferation and differentiation media.

### 2.5. Data Synthesis

Given the heterogeneity across experimental models, as well as fortification approaches, such as the type of fortificant used, time and duration of exposure, and reported outcomes, quantitative synthesis was not feasible. Instead, the extracted data were synthesized descriptively in both tabular and narrative formats to map the key characteristics of the included studies.

## 3. Results

### 3.1. Study Selection

**Figure 3:**
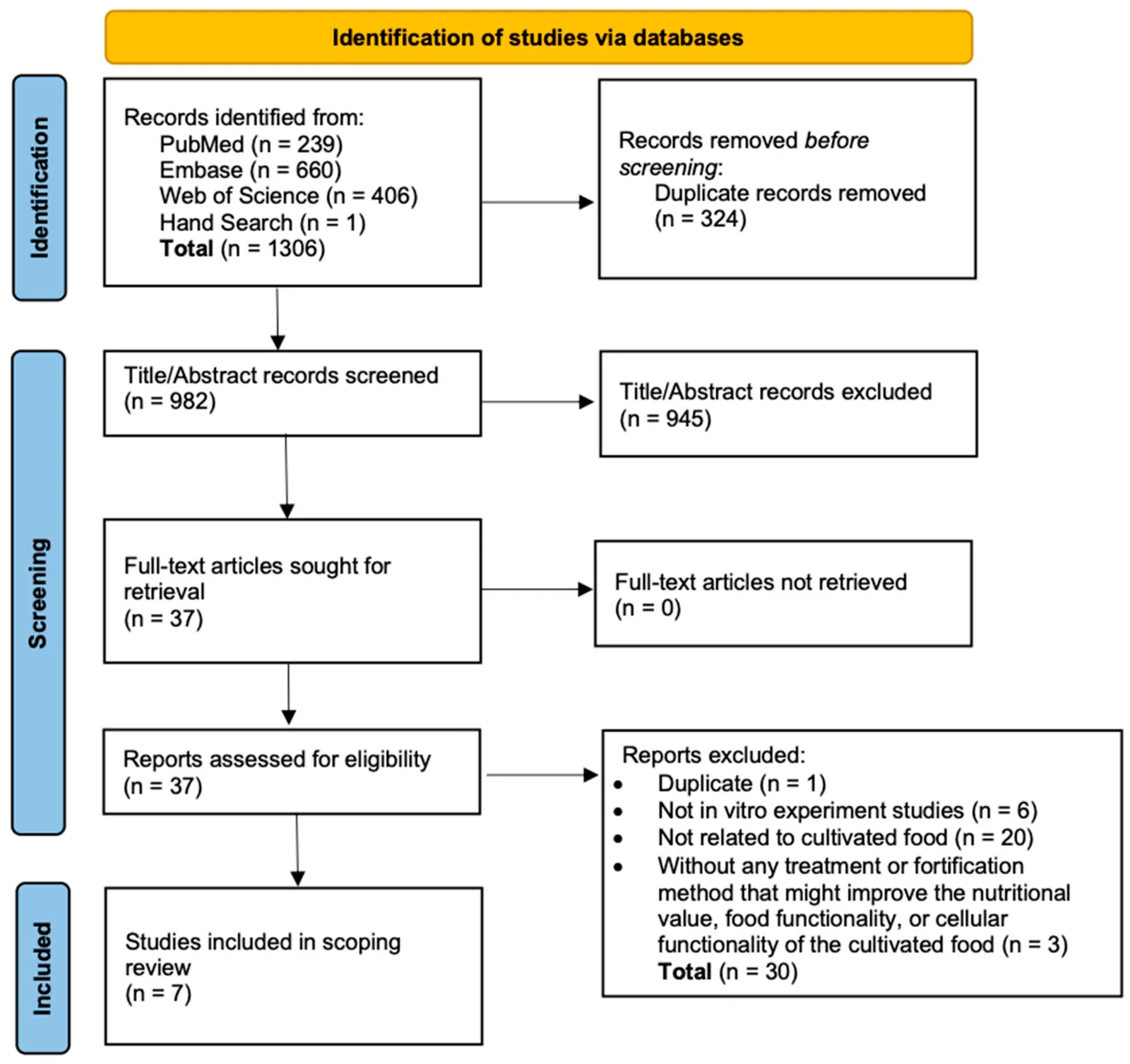
PRISMA flow diagram for study selection.

The PRISMA flow diagram for study selection is presented in Figure 3. A total of 1306 studies were identified from PubMed, Embase, Web of Science, and hand searching of relevant reference lists and key authors. Of the 1306 studies, 324 duplicates were found and excluded, followed by title and abstract screening of 982 studies, during which 945 studies were excluded. The full texts of the remaining 37 studies were sought for retrieval, and all of them were successfully retrieved. After the full text screening of 37 studies, 30 were excluded for different reasons, including ‘duplicate’ (*n* = 1), ‘not in vitro experiment studies’ (*n* = 6), ‘not related to CF’ (*n* = 20), and ‘without any treatment or fortification method that might improve the nutritional value or food functionality of the CF’ (*n* = 3). As a result, seven studies were eligible for inclusion in this scoping review.

### 3.2. Study Characteristics

The characteristics of the seven included studies are summarized in Table 1. The seven studies were published from 2020 to 2026, one in 2020 [19], one in 2022 [20], four in 2025 [21,22,23,24], and one in 2026 [25].

Regarding the cells used, two studies used C2C12 mouse cells [19,25], three used porcine cells, including fat cells [22], muscle stem cells [20] and dedifferentiated porcine cells [24], and two studies used bovine cells, including primary bovine satellite cells (BSC) [19] and adipose-derived mesenchymal stem cells [23] (Table 1). In addition, one study used Hanwoo-derived myosatellite cells (HSC) [21]. In relation to the passage number, cells were used in a wide range of passages. Zhu et al. [20] reported using P2–P15, Naseem et al. [23] using P2–P9, Sugama et al. [24] using P9–P24, Choi et al. [21] using P2, and Lanzoni et al. [25] using P9–P18. However, two studies did not report the passage number [19,22].

Most studies involved both cell proliferation and differentiation [19,20,21,22,23,24], except for Lanzoni et al. [25] which assessed only cell proliferation (Table 1). The proliferation duration ranged from 1 day to 8 days, whereas the differentiation duration ranged from 3 days to 8 days. However, Meng et al. [22] did not report both the duration of proliferation and differentiation, and Zhu et al. [20] did not specifically report the duration of proliferation.

### 3.3. Fortification Methods

There were three fortification methods, including cross-taxa metabolic engineering (*n* = 1) [19], product reformulation (i.e., incorporation of cultivated fat in food) (*n* = 1) [22], and enriched cell culture medium (*n* = 5) [20,21,23,24,25] (Table 1). The fortificants were obtained from chemical/biotechnology companies (*n* = 5) [19,20,23,24,25], research institute (*n* = 1) [21], or CM company (*n* = 1) [22] (Table S2).

#### 3.3.1. Metabolic Engineering

Stout et al. [19] applied metabolic engineering and reported outcomes related to nutritional value and food functionality. They introduced phytoene synthase (CrtB), phytoene desaturase (CrtI), and lycopene cyclase (CrtY) from the bacterium *Pantoea ananatis* into C2C12 and BSC at 80–90% confluence in 6-well plates through non-viral Sleeping Beauty transposon-mediated transgenesis, in which they found that the cells were able to undergo de novo carotenoid production and demonstrated significantly increased antioxidant activity (i.e., reduced lipid oxidation) in uncooked cells with lycopene and/or β-carotene during one day and eight days of refrigeration at 4 °C, as well as in cells cooked at 100 °C (*p* < 0.05) (Table 1).

#### 3.3.2. Product Reformulation

Meng et al. [22] reported outcomes related to nutritional value and sensory properties. They incorporated cultivated porcine fat cells into meatball formulations (Table 1). They found that the increase in the percentages of cultivated fats (10–30%) contributed significantly to the higher protein, fat, and total amino acids content of the meatballs (*p* < 0.05). Furthermore, the addition of the cultivated fat cells also significantly increased key contributors to a meat-like aroma, including total ketones and alcohols (*p* < 0.05). However, this also led to a significant reduction in total aldehydes, acids, and esters (*p* < 0.05).

#### 3.3.3. Enriched Cell Culture Medium

Zhu et al. [20] added L-ascorbic acid 2-phosphate (100 µM) to both proliferation and differentiation media (Table 1 and Table 2). Their results showed that the enriched cell culture media significantly improved cellular and food functionality by increasing the antioxidant capacity of the porcine muscle stem cells (*p* < 0.05) and improving cell proliferation (*p* < 0.05).

Naseem et al. [23] supplemented both proliferation and differentiation media with vitamin C (200 µM) and N-acetylcysteine (NAC) (2 mM), in which the addition of vitamin C (200 µM) or NAC (2 mM) alone or both significantly increased cell proliferation at 48 h, 72 h, and 96 h compared to the control group (*p* < 0.05). However, the addition of vitamin C also significantly improved cell differentiation while NAC did not significantly improve cell differentiation. Furthermore, both vitamin C and NAC also significantly reduced the reactive oxygen species (ROS) content in the cells (*p* < 0.05).

Choi et al. [21] added drone pupae extract (DEP) to the proliferation medium at concentrations ranging from 10 to 400 µg/mL. Then, a selected concentration of DEP was used in the differentiation medium (100 µg/mL). Their results demonstrated that DEP (10–400 µg/mL) significantly improved cell proliferation (*p* < 0.05) and increased cell viability through its antioxidant effect against H_2_O_2_-induced oxidative stress (*p* < 0.05). In addition, the addition of DEP during cell differentiation also improved myotube formation (*p* < 0.05).

Lanzoni et al. [25] added protein hydrolysates from hemp flowers (HF), hempseeds (HSS), hempseed protein (HP), shrimp (SH), or blue crab (BC) co-products to the proliferation medium at a concentration of 11.7 mg/mL, replacing the use of FBS. They found that the addition of SH co-products showed a comparable protein content in cell biomass compared to the 10% FBS control group (*p* > 0.05). Furthermore, the addition of HF, HSS, HP, SH, and BC protein hydrolysates demonstrated a significantly higher antioxidant activity in cell biomass and cell culture media compared to the 10% FBS control group (*p* ≤ 0.05).

Sugama et al. [24] added aroma precursors, including thiamine-HCl (500 µM), L-methionine (5.0 mM), or myoglobin (3.0 mg/mL), only to the differentiation medium for adipogenesis. Results showed that the addition of thiamine-HCl significantly increased the production of sulfurol, contributing to the milky aroma. Similarly, supplementing L-methionine significantly enhanced the production of methional (potato-like aroma). Furthermore, supplementing myoglobin significantly increased the concentration of alcohols, aldehydes, and furans, as well as aromas, which contributed to a coconut-like, deep fat, and fruity aroma (Table 1).

### 3.4. Cell Culture Media

The compositions of cell culture media used, including proliferation and differentiation media, in the studies are reported in Table 2. Regarding the proliferation medium, four studies used Dulbecco’s Modified Eagle Medium (DMEM) as the basal medium [19,23,24,25], two used F-10 medium (*n* = 2) [20,21], and one study reported the use of a proprietary serum-free medium [22]. Apart from Meng et al. [22], all other proliferation media were supplemented with fetal bovine serum (FBS), with proportions ranging from 10 to 20%, along with the use of antibiotics [19,20,21,23,24,25]. In addition, basic fibroblast growth factor (bFGF) was used as a supplement to support cell proliferation [19,20,21,24].

The differentiation media varied among studies due to different cell types involved (Table 2). For studies involving muscle cells, Stout et al. [19] used the same proliferation medium for differentiation, cultivating the cells to confluence and incubating them for one week without changing medium to induce myogenic differentiation. Zhu et al. [20] replaced 20% FBS in proliferation medium with 2% horse serum (HS) for differentiation, while Choi et al. [21] reduced the proportion of FBS from 20% to 2%. For studies involving adipocytes, Naseem et al. [23] replaced 10% FBS with 2% HS and added palmitoleic acid (100 μM) to the differentiation medium. On the other hand, Sugama et al. [24] used two different media for differentiation (i.e., induction medium and lipid accumulation medium). For the induction medium, supplements including insulin (3 μg/mL), 3-Isobutyl-1-methylxanthine (IBMX) (0.1 mM), dexamethasone (Dex) (0.3 μM), rosiglitazone (5 μM), biotin (10 μM), and calcium-D-pantothenate (5.67 μM) were used to induce differentiation. Furthermore, the lipid accumulation medium contained supplements, including rosiglitazone (5 μM), biotin (10 μM), L-ascorbic acid 2-phosphate trisodium salt (113 μM), and Intralipid (500 μg/mL). Whereas Meng et al. [22] used a proprietary serum-free medium for differentiation and Lanzoni et al. [25] did not perform differentiation.

## 4. Discussion

The purpose of this scoping review was to map and review the current evidence related to the strategies of nutritional fortification of CM, including metabolic engineering, product reformulation, and enriched cell culture medium. To our knowledge, it is the first study to systematically synthesize findings from in vitro studies, reporting the effects on cells with respect to nutritional value, food functionality, and cellular functionality through nutritional fortification. Overall, seven eligible studies were identified for inclusion, indicating a substantial research gap and highlighting the emerging nature of this research area.

The three fortification approaches identified in this review have different potential benefits, limitations, and practical applications. Although metabolic engineering may enable cells to synthesize or accumulate target compounds endogenously, thereby allowing fortification at the cellular level, this approach is technically complex and may raise additional safety and regulatory concerns [19]. Product reformulation may provide a more flexible and less complex approach to modify the nutritional composition of CM by incorporating the selected ingredients or cultivated tissues (e.g., fat tissue). However, this approach is performed at the final stage of production rather than during cell cultivation. Therefore, it may not have a direct influence on cellular functionality (i.e., cell proliferation, differentiation, and viability), although it may modify the food functionality of the final product. On the other hand, the enrichment of the cell culture medium is applied during cell proliferation and differentiation. Although it may provide benefits to nutritional value and food functionality, and may directly influence cell proliferation and differentiation, the extent of nutrient uptake from the medium and the accumulation of the fortificant in the harvested cells remain unclear.

The primary outcomes of the included studies varied, for example, some were from the perspective of improving the nutritional value of cultivated meat or fat [19,22,24,25], while some focused food and cellular functionality improvement, including sensory properties, antioxidant ability, cell proliferation and differentiation [20,21,23,24]. Despite the heterogeneity in study aims, the results of these studies suggested potential and preliminary approaches to improve nutritional value, food functionality and cellular functionality of CM which may be beneficial for the production of CM. Moreover, improved food functionality (i.e., antioxidant capacity) and cellular functionality (i.e., cell proliferation, differentiation, and viability) are particularly relevant because strategies to enhance CM yields involve either improving cell viability, minimizing cell death, or enhancing cell proliferation activity [26]. Although three included studies involved fortifying cell culture medium with micronutrients or other bioactive compounds [20,23,24], their effects on the nutrient composition of the resulting cell biomass were not directly assessed, requiring further investigation from a nutritional fortification perspective.

Conventional meat provides easily digestible proteins, amino acids, vitamins, and minerals [11]. Although some cultivated meats contain comparable amounts of protein and fat to those of conventional meat [9,27], the levels of vitamins and minerals in different types of CM have not been fully examined. More importantly, the nutritional value of raw and cooked CM has not been explored, as it is known that different cooking methods result in different degrees of loss of nutrients during the cooking process [28,29]. Therefore, it may be an important area for further investigation. This is particularly relevant for fortified CM because the nutritional benefits achieved through fortification may not necessarily be retained. Heat-sensitive vitamins (e.g., vitamins C and B) may be degraded [28,30], while cooking may also alter the bioavailability of other nutrients, such as iron [31]. Thus, the stability of the fortified nutrients during processing and cooking should also be taken into consideration, as ensuring that the intended nutritional benefits are maintained in the final product may represent an important practical challenge for CM fortification.

Added to the above is that conventional meat is one of the major sources of vitamins and minerals, such as vitamin B12, vitamin D_3_, and iron [32,33,34], which are essential to human health. The European Food Safety Authority (EFSA) has established dietary reference values (DRVs) for these micronutrients, setting adequate intakes (AIs) of 4 µg/day for vitamin B12 [35] and 15 µg/day for vitamin D [36] in adults, and a population reference intake (PRI) of 13 and 16 mg/day of iron in males and premenopausal females aged 18 or older, respectively [37]. Conventional meat contributes considerably to achieving these AIs and PRI because it provides highly bioavailable vitamin B12 and heme iron [32,38]. Therefore, if CM is to replace conventional meat, it should provide enough essential micronutrients to minimize potential health risks with this dietary transition. A similar situation can be observed in the dietary transition from animal-based to plant-derived foods, given that some micronutrients (e.g., vitamin B12) cannot be supplied by plants naturally [39], while some may have a lower bioavailability in plant-based diets (e.g., iron, zinc) [40]. However, the potential of CM should not necessarily be limited to reproducing the nutritional composition of conventional meat. Instead, CM may also provide opportunities for nutritional optimization, for example, by improving the fatty acid profile, increasing selected micronutrients, or enriching other beneficial compounds. Indeed, preliminary evidence suggests that nutritional optimization of CM may be feasible [41,42]. Therefore, future fortification strategies for CM should also consider nutritional optimization.

Although ensuring an adequate nutritional content in CM is essential, the bioavailability of the fortificants should also be considered. It is well established that the chemical form of a nutrient can significantly affect its bioavailability. For example, a substantial proportion (40–60%) of iron in conventional meat is heme iron, which generally has higher bioavailability than non-heme iron [43]. However, from the perspective of food fortification, non-heme iron compounds, including ferric pyrophosphate, ferrous fumarate, and ferrous sulfate, are commonly used as iron fortificants [43,44]. Although these non-heme iron compounds are generally less bioavailable than heme iron, they are effective in improving iron status [45]. However, as previously noted, the effects of food processing and cooking on nutrient retention and bioavailability in fortified CM remain unclear. Similar considerations should also apply to other micronutrients given that their chemical form may influence their stability and bioavailability. Therefore, further research is needed not only to evaluate the nutritional content of fortified CM but also to assess the chemical form, bioaccessibility, and bioavailability of the nutrients in the final product.

Food fortification can be broadly divided into two categories: direct fortification and biofortification [46,47]. Direct fortification involves adding desired fortificants directly to the food during processing, and this strategy has been widely used in the fortification of, for example, dairy products and cereals [48]. Similarly, fortification of CM through cell culture medium also involves direct addition of fortificants during cell cultivation (i.e., proliferation and differentiation). This assumes that cells used for CM production have the potential to utilize and store nutrients from the cell culture media to achieve nutrient fortification. However, there is limited evidence on whether CM could take up vitamins and minerals from enriched culture medium, and whether these would be bioaccessible to consumers [49]. The uncertainty regarding the fortificant accumulation may partly stem from the fundamental differences between in vivo and in vitro environments. In vivo, nutrients are absorbed and accumulated through a sequence of biological processes [15]. However, in vitro culture does not reproduce whole-animal processes such as gastrointestinal absorption and organ-specific nutrient storage, indicating that increasing concentration of a nutrient in the medium may not necessarily result in accumulation in the harvested cells. One potential approach to overcome this limitation may be the use of genetic or metabolic engineering of nutrient uptake and storage pathways. For example, Narayanan et al. [50] used genetic engineering to improve iron and zinc accumulation in cassava by co-expressing the iron transporter (IRT1) and the iron-storage protein ferritin (FER1) derived from *Arabidopsis thaliana*. However, further investigation is required to determine the feasibility of applying a similar strategy to CM production.

As reported in studies related to enriched cell culture media, enriched cell culture media may contribute to the nutritional value and metabolites related to sensory characteristics of the cultivated meat or fat [24,25]. Additionally, positive effects on cell proliferation, differentiation, and antioxidant ability were observed [20,21,23,24,25]. However, it is worth noting that these studies were conducted on small-scale experiments over short-term proliferation and differentiation duration and using cell line models. Therefore, the findings may not be generalizable to large-scale industrial production settings, in which long-term proliferation and differentiation may be required, given that the overall cultivated meat production may take approximately 3–4 weeks [51]. Moreover, cellular behavior may also differ considerably over long-term proliferation and differentiation [26,52]. Nevertheless, in vitro models suggested that enriched cell culture media could provide benefits to cell cultivation, including improved proliferation and differentiation, nutritional value and antioxidant capacity.

On the other hand, biofortification describes the process of breeding nutrients into food crops, which could potentially provide more micronutrients [48,53]. However, more research has been conducted on animals recently, for example, vitamin D_3_ supplementation in hens and cows [54,55] and ultraviolet-B exposure to dairy cows [56]. These studies aimed to fortify animal-derived products (e.g., eggs and milk) by improving the nutritional status of the animals. Likewise, in relation to CM, this scoping review identified one paper regarding metabolic engineering [19], in which BSCs and C2C12 cells were able to produce phytonutrients endogenously, including carotenoids, phytoene, lycopene, and β-carotene. These phytonutrients offer health benefits such as reducing the risk of cancer [19,57], demonstrating that cultivated meat could potentially be healthier than conventional meat products, especially red and processed meat that has been associated with increased risk of cancer [58]. However, it is important to note that the definitions of direct fortification and biofortification have not yet been clearly established in the context of CM, given that both concepts were originally developed in the context of conventional food, representing a conceptual gap that requires further investigation.

It may have implications for future regulatory development, given that CM is now classified as a novel food in the European Union (EU) [59]. Specifically, the safety of such products for human consumption would be assessed within the scope of the novel food framework before market authorization, including considerations related to toxicity, allergenicity, production process, and nutritional characteristics [60]. In this review, three preliminary approaches were identified that may modify the nutritional value, food functionality, or cellular functionality of CM. However, these approaches may require different safety assessments and regulatory considerations depending on their biological mechanisms and stages of application. For genetic engineering, additional regulatory considerations may be required given the involvement of genetic modification. Therefore, the resulting product may fall within the scope of genetically modified food and feed (EC No. 1829/2003) [61]. For cell culture medium enrichment, safety assessments may include the concentrations of fortificants, medium components, as well as the potential residues or accumulation in the harvested cells [62]. In contrast, product reformulation primarily occurs during the food-processing and formulation stage, modifying the composition of the final CM product. Therefore, in addition to the considerations related to CM itself, the incorporated ingredients should also meet the safety and regulatory requirements.

Apart from the fortification strategies identified in this scoping review, it is also worth mentioning that incorporating edible scaffolds into CM could be a strategy to improve the nutritional value of CM products. However, edible scaffolding is fundamentally different from food fortification. The main purpose of scaffolding in CM is to provide a three-dimensional (3D) structure that mimics the extracellular matrix of conventional meat [63,64], although scaffolds containing biomaterials could also offer nutritional benefits [65]. In fact, some studies have explored the use of plant-based proteins, such as pea and soy protein, to create edible 3D scaffolds in the context of cultivated meat production [66,67]. These plant proteins are sustainable and high-quality protein sources [68,69], but the risk of allergy should also be taken into consideration, given that both pea and soy proteins are allergenic [70,71]. Nevertheless, the development of nutritionally optimized edible scaffolds may represent a complementary future strategy for enhancing the nutritional value of CM.

As widely reported in the literature, one of the critical challenges of CM production is the replacement of FBS [7,49,72,73]. FBS has been used in cell cultivation for a long time owing to its rich and complex composition, which provides essential components for cell proliferation, such as hormones, vitamins, transport proteins, and growth factors [74,75,76]. However, the unethical production of FBS and concerns about biosafety highlight the need to reduce and replace FBS [77], especially in the context of human consumption. It is important to mention that FBS is considered an animal byproduct, which is not intended for human consumption under the EU regulation (No. 1069/2009) [78]. One of the included studies also investigated the replacement of FBS in addition to nutritional fortification [25], although most of the included studies used different percentages of FBS (10–20%) or HS (Table 2). Moreover, another study used a serum-free culture medium provided by a CM company [22]. In fact, there is already some evidence on the replacement of FBS with alternative protein sources, for example, plant- and dairy-derived proteins, demonstrating their ability to support cell proliferation [6,79,80,81] and also differentiation in serum-reduced medium [82,83]. Notably, Stout et al. [84] and Stout et al. [85] developed serum-free culture media capable of supporting the proliferation and differentiation of muscle stem cells, while Mitić et al. [86] demonstrated the same for cultivated fat. However, it is worth noting that the use of FBS may represent a confounding factor, given that FBS is a complex and chemically undefined supplement containing various bioactive compounds, including vitamins, that may interact with the fortificants. Therefore, to better isolate and validate the effects of the fortificants, the use of chemically defined serum-free media is recommended in future studies.

Regarding the strength of the current evidence, the findings of the seven included studies should be considered preliminary. Although the investigated treatments may have beneficial effects on the production of CM, substantial heterogeneity was observed in study designs, treatment conditions, primary outcomes, cell models and culture media. Several studies used research-oriented cell models (e.g., C2C12 and BSCs) rather than models specifically developed for CM production, which may limit the transferability of the findings to large-scale production systems. Moreover, most studies focused on cellular functions such as proliferation and differentiation, or antioxidant capacity rather than directly evaluating the nutritional value of CM. The use of serum-containing culture media (i.e., FBS and HS) or proprietary media may further reduce the applicability of the findings to food-grade CM production.

This scoping review has several strengths and limitations. To our knowledge, it is the first scoping review to systematically map the evidence on nutritional fortification strategies for CM. This review was conducted in accordance with the PRISMA-ScR and JBI guidance. In addition, this review provides a comprehensive and structured overview of the fortification methods, cell types, culture media, and reported nutritional and functional outcomes, which may help to identify key research gaps in this emerging research area. Nevertheless, several limitations should be acknowledged. Given the novelty of this topic, only seven eligible studies were identified. Moreover, the included studies were highly heterogeneous in terms of cell types, culture media, and outcome measures. It is also worth mentioning that the cells used in the included studies such as C2C12 and BSCs were primarily intended for research purposes rather than human consumption. Additionally, most studies did not directly assess the nutritional profile of the CM but instead focused on food functionality such as antioxidant capacity. Therefore, the effects of fortification on the nutritional profile of CM, including both macronutrients and micronutrients, remain unclear, highlighting a research gap that requires further investigation.

## 5. Conclusions

Given the continued growth of the global population and the increasing demand for meat products, CM may represent a potentially sustainable alternative to conventional meat. However, one of the critical challenges is ensuring that the nutritional value of CM is comparable to that of conventional meat, including both macronutrients and micronutrients. This scoping review systematically synthesized evidence from in vitro studies and identified several potential nutritional fortification approaches, including metabolic engineering, product reformulation, and nutrient-enriched cell culture medium. Among these approaches, metabolic engineering showed potential to improve the nutritional value and antioxidant-related properties of CM. Product reformulation may modify nutritional value and sensory properties of CM, whereas nutrient-enriched media may improve cellular functionality, including cell proliferation and differentiation. However, it is worth noting that most of the included studies were not primarily designed to assess post-fortification nutritional profiles, instead focusing on antioxidant capacity or effects on cell proliferation and differentiation. Although their findings provide preliminary evidence for potential fortification strategies for CM, future research aimed at investigating CM fortification and its effects on the nutritional profile of the resulting cell biomass or final CM product is needed.

## Figures and Tables

**Table 1:** Characteristics of the seven included in vitro studies.

Author (Year)	Cell Type (Passage Number)	Proliferation Duration; Differentiation Duration	Fortification Method	Primary Outcome	Control Group	Findings
Stout et al. (2020) [19]	1. C2C12 cells (NA) 2. BSC (NA)	8 days; 7 days	Cross-taxa metabolic engineering: Phytoene synthase (CrtB), phytoene desaturase (CrtI), and lycopene cyclase (CrtY) from the bacterium *Pantoea ananatis* were introduced to C2C12 cells and BSC using non-viral Sleeping Beauty transposon-mediated transgenesis.	Endogenous carotenoid production for nutritional enhancement	Cells transfected with the pGFP control vector	De novo production of carotenoid: pCrtB cells: phytoene pCrtB/I cells: phytoene and lycopene pCrtB/I/Y cells: phytoene, lycopene and β-carotene Reduced lipid oxidation: ↑ Antioxidant effects in cells with lycopene and/or β-carotene
Zhu et al. (2022) [20]	Porcine muscle stem cells isolated from a 1-week-old pig (P2–15)	Passage every 3 days; 3–6 days	Enriched culture media: 100 µM L-ascorbic acid 2-phosphate was added to proliferation and differentiation media.	Maintenance of cellular functions during cell expansion and long-term culture	Standard culture medium without Asc-2P supplementation	Addition of 100 µM L-ascorbic acid 2-phosphate: ↑ Antioxidant capacity (measured at one day of proliferation) ↑ Proliferation (*p* < 0.05) (measured after 3 days of proliferation)
Meng et al. (2025) [22]	Porcine fat cells (NA)	NA	Product reformulation: 10–30% of cultivated fat cells were mixed with other ingredients to produce the meatball, including 20% soy protein, 20% tapioca starch, 3% NaCl, 1.8% carboxymethyl cellulose, and 0.2% sodium tripolyphosphate.	Investigation of the effects of varying proportions of cultivated fat biomass on the flavor and nutritional characteristics of meatballs	BPM-0%: No fat biomass (replaced with 55% water). FPM-10%: Reference group using 10% pig subcutaneous fat	Addition of cultivated fat: ↑ Protein, fat, and total amino acid content of the meatball (*p* < 0.05) ↑ Total ketones and alcohols (key contributors to a meat-like aroma) (*p* < 0.05) ↓ Total aldehydes, acids, and esters decreased (*p* < 0.05)
Naseem et al. (2025) [23]	Bovine adipose-derived mesenchymal stem cells extracted from the adipose tissues of Yanbian cattle testicles (P2–9)	4 days; 4 days	Enriched culture media: NAC (2 mM) and VC (200 µM) were added to the proliferation and differentiation media	To assess the effects of VC and NAC supplementation on the proliferation and differentiation capacities of bovine adipose-derived mesenchymal stem cells	No antioxidants (VC or NAC) added	Addition of NAC (2 mM): ↑ Proliferation (*p* < 0.05) compared to control at 48 h, 72 h, and 96 h ~Differentiation (*p* > 0.05) compared to control (measured at 96 h) ↓ ROS content (*p* < 0.05) compared to control Addition of vitamin C (200 µM): ↑ Proliferation (*p* < 0.05) compared to control at 48 h, 72 h, and 96 h ↑ Differentiation (*p* < 0.05) compared to control (measured at 96 h) ↓ ROS content (*p* < 0.05) compared to control Addition of NAC and vitamin C (2 mM and 200 µM): ↑ Proliferation (*p* < 0.05) compared to control at 48 h, 72 h, and 96 h ↑ Differentiation (*p* < 0.05) compared to control (measured at 96 h) ↓ ROS content (*p* < 0.05) compared to control
Sugama et al. (2025) [24]	pDFAT (P9–24)	1–5 days; 8 days	Enriched culture media: Adipogenic differentiation media with the addition of thiamine-HCl (500 µM) and L-methionine (5.0 mM) from day 2 to 8 ^a^. Myoglobin (3.0 mg/mL) was added 24 h before cell harvest.	To modulate aroma-related volatile compounds in heated porcine fat cells	No aroma precursors (Thiamine-HCl, L-methionine, or Myoglobin) added	Addition of thiamine-HCl: ↑ Sulfurol (milky aroma) (*p* < 0.001) ~ thiamine and thiamine pyrophosphate (NS) ^c^ Addition of L-methionine: ↑ Methional (potato-like aroma) (*p* < 0.001) ~ Methionine and methionine sulfoxide (NS) ^c^ Addition of myoglobin: ↑ Aroma precursors ^b^ ↑Alcohols, aldehydes and furans (*p* < 0.05)
Choi et al. (2025) [21]	HSC (P2)	5 days; 3–4 days	Enriched culture media: 10, 100, 200, 400 µg/mL of drone pupae extract powder (DEP) was added to the proliferation media. DEP at 0 and 100 µg/mL concentrations were added to the differentiation media.	To examine the effects of DEP supplementation on the proliferation and differentiation of HSCs	No DEP added to medium	Addition of DEP (10–400 µg/mL) during proliferation: ↑ Cell viability due to the antioxidant effect against H_2_O_2_-induced oxidative stress (*p* < 0.05) ↑ Proliferation (*p* < 0.05) Addition of DEP (100 µg/mL) during differentiation: ↑ Myotube formation (*p* < 0.05)
Lanzoni et al. (2026) [25]	C2C12 cells (P9–18)	3 days; Not performed	Enriched culture media: Protein hydrolysates stemmed from hemp flowers (HF), hempseeds (HSS), hempseed protein (HP), shrimp (SH), or blue crab (BC) co-products were supplemented at concentrations of 11.7 mg/mL to culture media, replacing FBS	Investigate protein hydrolysates as sustainable culture media supplements	Negative Control: 0% FBS Positive Control: 10% FBS	Addition of 11.7 mg/mL of HF, HSS, HP, SH, or BC: ~ Similar protein content of cell biomass for SH compared to 10% FBS (*p* > 0.05) ↑ Antioxidant activity in cell biomass and cell culture media compared to 10% FBS (*p* ≤ 0.05)

BSC, bovine satellite cell; pDFAT, Porcine dedifferentiated fat cells; GGPP, geranylgeranyl pyrophosphate; MUFA, monounsaturated fatty acid; PUFA, polyunsaturated fatty acid; NS, non-significance; HSC, Hanwoo myosatellite cells; DEP, drone pupae extract powder; HF, hemp flowers; HSS, hempseeds; HP, hempseed protein; SH, shrimp; BC, blue crab; FBS, fetal bovine serum; VC, vitamin C; NAC, N-acetylcysteine; BPM, biomass/plant hybrid cultured meatball; FPM, pork fat meatball; NA, not available. The signs ↑, ↓, and ~ denote a significant increase, decrease, and non-significant difference, respectively. ^a^ Day 0 was defined as the day of media transition from proliferation media to adipogenic differentiation media. ^b^ γ-nonalactone (coconut-like aroma, *p* < 0.01), (E, E)-2,4-decadienal (deep fat aroma), 2-pentylfuran (fruity aroma), δ-decalactone (peachy aroma), benzeneacetaldehyde (honey aroma), heptanal (green and fatty aroma) and 1-pentanol (sweet aroma) (*p* < 0.05). ^c^ Intracellular accumulation from enriched cell culture media.

**Table 2:** Components of the cell culture media used in the seven included studies.

Study	Serum-Free/-Based Medium	Proliferation Medium	Differentiation Medium
[19]	Serum-based	C2C12: DMEM Glutamax 10% FBS 1% antibiotic–antimycotic BSC: DMEM Glutamax 20% FBS human FGF-basic 1 ng/mL 1% Primocin ^a^	C2C12: Same as proliferation medium ^b^ BSC: Same as proliferation medium ^b^
[20]	Serum-based	F10 medium 20% FBS bFGF (5 ng/mL) 1% P/S	DMEM 2% horse serum 1% P/S
[22]	Serum-free	Proprietary serum-free medium by Nanjing Joes Future Food Technology Co., Ltd, Nanjing, China	Proprietary serum-free medium by Nanjing Joes Future Food Technology Co., Ltd.
[23]	Serum-based	DMEM 10% FBS 1% PSG	DMEM 2% horse serum 1% PSG Palmitoleic acid (100 μM)
[24]	Serum-based	DMEM GlutaMax 15% FBS Primocin (100 μg/mL) bFGF (10 ng/mL)	Induction Media: DMEM GlutaMax 10% FBS Primocin (100 μg/mL) Insulin (3 μg/mL) IBMX (0.1 mM) Dexamethasone (0.3 μM) Rosiglitazone (5 μM) Biotin (10 μM) Calcium-D-pantothenate (5.67 μM) Lipid Accumulation Media: DMEM GlutaMax Primocin (100 μg/mL) Rosiglitazone (5 μM) Biotin (10 μM) L-ascorbic acid 2-phosphate trisodium salt (113 μM) Intralipid (500 μg/mL)
[21]	Serum-based	Ham’s F-10 medium 20% FBS 1% PSA 0.05% bFGF	DMEM 2% FBS 1% PSA
[25]	Serum-based	High-glucose DMEM 10% FBS ^c^ 2 mM L-glutamine 1% PSA	Not performed

DMEM, Dulbecco’s Modified Eagle Medium; FBS, fetal bovine serum; BSC, bovine satellite cell; bFGF, basic fibroblast growth factor; P/S, penicillin–streptomycin; IBMX, 3-Isobutyl-1-methylxanthine; PSG, penicillin–streptomycin–gentamicin; PSA, penicillin–streptomycin–amphotericin B. ^a^ After two weeks of cell culture, primocin was replaced with 1% antibiotic–antimycotic. ^b^ Cells were cultured to confluence and then incubated for one week without changing medium to induce myogenic differentiation. ^c^ FBS was replaced during treatment.

## Data Availability

The original contributions presented in this study are included in the article/Supplementary Materials. Further inquiries can be directed to the corresponding author.

## Supplementary Materials

The following supporting information can be downloaded at: https://www.mdpi.com/article/10.3390/foods15162840/s1, Table S1: Preferred Reporting Items for Systematic Reviews and Meta-Analyses extension for Scoping Reviews (PRISMA-ScR) Checklist; Method S1: Search strategies; Table S2: Keywords applied for literature search on PubMed, Web of Science, and Embase; Method S2: Sources of fortificants; Table S3: Sources of fortificants used in the included studies.
