# Supplementary material for: Nutritional and Functional Fortification of Cultivated Meat: A Scoping Review
Source: Foods. 2026 Aug 14;15(16):2840. doi: 10.3390/foods15162840 (PMC13512501; doi:10.3390/foods15162840)
Supplement: Supplementary file 1 [file foods-15-02840-s001.zip › foods-4452099-supplementary.pdf]

Supplementary Materials

Table S1. Preferred Reporting Items for Systematic Reviews and Meta-Analyses Extension for Scoping Reviews (PRISMA-ScR) Checklist

SECTION	ITEM	PRISMA-ScR CHECKLIST ITEM	REPORTED ON PAGE #
**TITLE**			
Title	1	Identify the report as a scoping review.	1
**ABSTRACT**			
Structured summary	2	Provide a structured summary that includes (as applicable): background, objectives, eligibility criteria, sources of evidence, charting methods, results, and conclusions that relate to the review questions and objectives.	2
**INTRODUCTION**			
Rationale	3	Describe the rationale for the review in the context of what is already known. Explain why the review questions/objectives lend themselves to a scoping review approach.	3-6
Objectives	4	Provide an explicit statement of the questions and objectives being addressed with reference to their key elements (e.g., population or participants, concepts, and context) or other relevant key elements used to conceptualize the review questions and/or objectives.	6
**METHODS**			
Protocol and registration	5	Indicate whether a review protocol exists; state if and where it can be accessed (e.g., a Web address); and if available, provide registration information, including the registration number.	7
Eligibility criteria	6	Specify characteristics of the sources of evidence used as eligibility criteria (e.g., years considered, language, and publication status), and provide a rationale.	7
Information sources*	7	Describe all information sources in the search (e.g., databases with dates of coverage and contact with authors to identify additional sources), as well as the date the most recent search was executed.	7-8
Search	8	Present the full electronic search strategy for at least one database, including any limits used, such that it could be repeated.	7-8, Supplementary material
Selection of sources of evidence†	9	State the process for selecting sources of evidence (i.e., screening and eligibility) included in the scoping review.	8
Data charting process‡	10	Describe the methods of charting data from the included sources of evidence (e.g., calibrated forms or forms that have been tested by the team before their use, and whether data charting was done independently or in duplicate) and any processes for obtaining and confirming data from investigators.	8
Data items	11	List and define all variables for which data were sought and any assumptions and simplifications made.	8
Critical appraisal of individual sources of evidence§	12	If done, provide a rationale for conducting a critical appraisal of included sources of evidence; describe the methods used and how this information was used in any data synthesis (if appropriate).	Not applicable
Synthesis of results	13	Describe the methods of handling and summarizing the data that were charted.	8
**RESULTS**			
Selection of sources of evidence	14	Give numbers of sources of evidence screened, assessed for eligibility, and included in the review, with reasons for exclusions at each stage, ideally using a flow diagram.	9
Characteristics of sources of evidence	15	For each source of evidence, present characteristics for which data were charted and provide the citations.	10-16
Critical appraisal within sources of evidence	16	If done, present data on critical appraisal of included sources of evidence (see item 12).	Not applicable
Results of individual sources of evidence	17	For each included source of evidence, present the relevant data that were charted that relate to the review questions and objectives.	10-16
Synthesis of results	18	Summarize and/or present the charting results as they relate to the review questions and objectives.	14-16
**DISCUSSION**			

SECTION	ITEM	PRISMA-ScR CHECKLIST ITEM	REPORTED ON PAGE #
Summary of evidence	19	Summarize the main results (including an overview of concepts, themes, and types of evidence available), link to the review questions and objectives, and consider the relevance to key groups.	17-21
Limitations	20	Discuss the limitations of the scoping review process.	21
Conclusions	21	Provide a general interpretation of the results with respect to the review questions and objectives, as well as potential implications and/or next steps.	22
**FUNDING**			
Funding	22	Describe sources of funding for the included sources of evidence, as well as sources of funding for the scoping review. Describe the role of the funders of the scoping review.	Not applicable

JBI = Joanna Briggs Institute; PRISMA-ScR = Preferred Reporting Items for Systematic reviews and Meta-Analyses extension for Scoping Reviews.

* Where *sources of evidence* (see second footnote) are compiled from, such as bibliographic databases, social media platforms, and Web sites.

† A more inclusive/heterogeneous term used to account for the different types of evidence or data sources (e.g., quantitative and/or qualitative research, expert opinion, and policy documents) that may be eligible in a scoping review as opposed to only studies. This is not to be confused with *information sources* (see first footnote).

‡ The frameworks by Arksey and O'Malley (6) and Levac and colleagues (7) and the JBI guidance (4, 5) refer to the process of data extraction in a scoping review as data charting.

§ The process of systematically examining research evidence to assess its validity, results, and relevance before using it to inform a decision. This term is used for items 12 and 19 instead of "risk of bias" (which is more applicable to systematic reviews of interventions) to include and acknowledge the various sources of evidence that may be used in a scoping review (e.g., quantitative and/or qualitative research, expert opinion, and policy document).

Method S1. Search strategies

Table S2. Keywords applied for literature search on PubMed, Web of Science, and Embase.

Step No.	Keywords	Results
	Database: PubMed	
1	"Vitamins"[Mesh] OR "Vitamin*"[All Fields] OR "Micronutrients"[Mesh] OR "Micronutrient*"[All Fields] OR "Nutrients"[Mesh] OR "Nutrient*"[All Fields]	610250
2	"fortification"[All Fields] OR "fortification*"[All Fields] OR "food, fortified"[MeSH Terms] OR "Biofortification"[Mesh] OR “biofortif*”[All Fields] OR “enrich*”[All Fields] OR “enhance*”[All Fields]	2662916
3	"In Vitro Meat"[Mesh] OR "cultivated meat*"[All Fields] OR "cellular meat*"[All Fields] OR "cultivated food*"[All Fields] OR "cellular food*"[All Fields] OR "lab-grown"[All Fields] OR "Novel food*"[All Fields] OR "cell-based"[All Fields] OR "cellular agriculture"[All Fields]	57401
4	#1 AND #2 AND #3	239
	Database: Web of Science	
1	ALL=("Vitamin*" OR "Micronutrient*” OR "Nutrient*")	994875
2	ALL=("fortif*" OR “biofortif*” OR “enrich*” OR “enhance*”)	5761091
3	ALL=("In Vitro Meat"OR "cultivated meat*" OR "cellular meat*" OR "cultivated food*" OR "cellular food*" OR "lab-grown" OR "Novel food*"OR "cell-based" OR "cellular agriculture")	76531
4	#1 AND #2 AND #3	406
	Database: Embase	
1	'vitamin'/exp OR 'vitamin*' OR 'trace element'/exp OR 'micronutrient*' OR 'nutrient'/exp OR 'nutrient*'	1346117
2	'fortification' OR 'fortification*' OR 'fortified food'/exp OR 'biofortification'/exp OR 'biofortif*' OR 'enrich*' OR 'enhance*'	3436090
3	'cultured meat'/exp OR 'cultivated meat*' OR 'cellular meat*' OR 'cultivated food*' OR 'cellular food*' OR 'lab-grown' OR 'novel food*' OR 'cell-based' OR 'cellular agriculture'	78321
4	#1 AND #2 AND #3	660

Method S2. Sources of fortificants

Table S3. Sources of fortificants used in the included studies.

Study	Fortificant	Source of fortificant
Stout et al. (2020) [5]	Amino acid sequences for *CrtB, Crt*I, and *CrtY*	*Pantoea ananatis*
Zhu et al. (2022) [7]	L-ascorbic acid 2-phosphate	Chemical company
Meng et al. (2025) [3]	Porcine fat biomass	Cultivated food company
Naseem et al. (2025) [4]	Vitamin C and N-acetylcysteine	Chemical company
Sugama et al. (2025) [6]	Thiamine-HCl, L-methionine, and myoglobin	Chemical company
Choi et al. (2025) [1]	Drone pupae extract	Research institute
Lanzoni et al. (2026) [2]	Hemp flowers (HF), hempseeds (HSs), hempseed protein (HP), shrimp (SH), and blue crab (BC)	HF: company<br>HS: company<br>HP: company<br>SH: *Penaeus spp*<br>BC: *Callinectes sapidus*

References

1. Choi, N., Park, S., Park, G., Oh, S., Lee, S. H., Lee, J., Kim, H., Bang, G., and Choi, J. (2025). Drone pupae extract enhances Hanwoo myosatellite cell function for cultivated meat production. J Anim Sci Technol, 67(1), 252–272. https://doi.org/10.5187/jast.2024.e98
2. Lanzoni, D., Manuguerra, S., Arena, R., Santulli, A., Marchetti, L., Messina, C. M., & Giromini, C. (2026). Valorization of Hemp, Shrimp and Blue Crab Co-Products as Novel Culture Media Ingredients to Improve Protein Quality and Antioxidant Capacity of Cultured Meat in Cell-Based Food Applications. Foods, 15(2), 352.
3. Meng, Z. Q., Tang, C. B., Tao, T. Y., Song, W., Li, C. B., Qi, J., Ding, S. J., and Zhou, G. H. (2025). Cultured biomass enhances the flavor and nutrition characteristics of biomass/plant hybrid cultured meatballs. Food Res Int, 214, 116627. https://doi.org/10.1016/j.foodres.2025.116627
4. Naseem, S., Xuan, M. F., Hua, H., Park, S., Manzoor, A., Teng, H., Jin, H., Li, X., & Li, Q. (2025). Vitamin C and N-acetylcysteine promote bovine adipose-derived mesenchymal stem cell proliferation and differentiation via Akt/mTOR/P70S6K signalling pathway for cultured meat production. Anim Biosci, 38(10), 2250–2265. https://doi.org/10.5713/ab.24.0776
5. Stout, A. J., Mirliani, A. B., Soule-Albridge, E. L., Cohen, J. M., & Kaplan, D. L. (2020). Engineering carotenoid production in mammalian cells for nutritionally enhanced cell-cultured foods. Metabolic Engineering, 62, 126–137. https://doi.org/10.1016/j.ymben.2020.07.011
6. Sugama, N., Lew, E. T., Riquelme-Guzmán, C., Lee, D. S., Li, X., Yuen, J. S. K., Lim, T., Kwan, A., Liu, R. Y., Ma, Y. A., Frost, S. C., & Kaplan, D. L. (2025). Modulation of nutritional composition and aroma volatiles in cultivated pork fat by culture media supplementation. Frontiers in Nutrition, Volume 12 - 2025. https://doi.org/10.3389/fnut.2025.1674183
7. Zhu, H., Wu, Z., Ding, X., Post, M. J., Guo, R., Wang, J., Wu, J., Tang, W., Ding, S., & Zhou, G. (2022). Production of cultured meat from pig muscle stem cells. Biomaterials, 287. https://doi.org/10.1016/j.biomaterials.2022.121650
